# Percutaneous intragastric approach for endoscopic hand suturing of the defect after resection of a submucosal gastric tumor

**DOI:** 10.1055/a-2653-9173

**Published:** 2025-08-22

**Authors:** Przemysław Kasprzyk, Zofia Orzeszko, Kasper Maryńczak, Urszula Zawada, Michal Spychalski

**Affiliations:** 1 Endoscopy and Bariatric Surgery Center, Brzeziny Specialist Hospital, Brzeziny, Poland; 2 37799 Department of Surgery, Jagiellonian University, Kraków, Poland; 3 37808 Department of General and Oncological Surgery, Medical University of Lodz, Lodz, Poland

The role of endoscopic resection in the management of subepithelial lesions (SELs) of the stomach is expanding 1 . The complexity of the procedure depends on the tumor’s location, size, and depth of invasion. Lesions situated in the subcardia present distinct technical challenges, particularly with respect to closure of the resulting wall defect 2 . Endoscopic hand suturing (EHS) has been demonstrated to be a feasible technique for closure of various defects following endoscopic interventions 3 . In this report we present a novel method of percutaneous intragastric EHS (PI-EHS) for managing defects in the gastric subcardia.

A 70-year-old woman was admitted with a 35-mm SEL located in the gastric subcardia. Endoscopic ultrasonography revealed tumor infiltration into the muscular layer, so the patient was scheduled for laparoscopic endoscopic cooperative surgery 4 . Initially, we proceeded with endoscopic dissection; however, owing to difficulties in tumor mobilization, a full-thickness resection was performed, resulting in a transmural defect that required closure. Conventional closure with the endoscope in the retroflexed position was technically challenging, which prompted a decision to adopt a percutaneous intragastric approach. Under endoscopic guidance, a laparoscopic trocar was inserted directly into the gastric lumen. Subsequently, a gastroscope was introduced through the trocar, facilitating an antegrade position from which to perform EHS for defect closure ( Video 1 ).

During laparoscopic endoscopic cooperative surgery, a large subepithelial lesion was managed by full-thickness resection. Owing to the location of the defect, endoscopic hand suturing was performed via an intragastric trocar inserted by the surgeon under endoscopic guidance, which facilitated suturing with the endoscope in an antegrade position.Video 1

The postoperative period was uneventful, and a follow-up endoscopy on postoperative day (POD) 2 confirmed the intact suture line. A course of antibiotics (ciprofloxacin and metronidazole) was administered, along with proton pump inhibitors. A liquid diet was allowed on POD3, and advanced to a solid diet after 1 week. The patient was discharged in good condition on POD4 and remained well at the 30-day follow-up with no complaints. Pathological examination subsequently revealed a leiomyoma.

This case exemplifies the efficient use of PI-EHS for the management of a large defect following full-thickness resection in the gastric subcardia.

Endoscopy_UCTN_Code_TTT_1AO_2AO

## Notes

Endoscopy E-Videos https://eref.thieme.de/e-videos
*E-Videos* is an open access online section of the journal *Endoscopy* , reporting on interesting cases and new techniques in gastroenterological endoscopy. All papers include a high-quality video and are published with a Creative Commons CC-BY license. Endoscopy E-Videos qualify for HINARI discounts and waivers and eligibility is automatically checked during the submission process. We grant 100% waivers to articles whose corresponding authors are based in Group A countries and 50% waivers to those who are based in Group B countries as classified by Research4Life (see: https://www.research4life.org/access/eligibility/ ). This section has its own submission website at https://mc.manuscriptcentral.com/e-videos .
